# Amyotrophic lateral sclerosis and retinal changes in optical coherence tomography: A systematic review and meta‐analysis

**DOI:** 10.1002/brb3.2741

**Published:** 2022-08-22

**Authors:** Gaurav Nepal, Sanjeev Kharel, Megan Ariel Coghlan, Jayant Kumar Yadav, Pawan Parajuli, Kamal Pandit, Yow Ka Shing, Rajeev Ojha

**Affiliations:** ^1^ Department of Internal Medicine Maharajgunj Medical Campus Tribhuvan University Institute of Medicine Maharajgunj Kathmandu Nepal; ^2^ Department of Neurology University of Louisville School of Medicine Louisville Kentucky US; ^3^ Department of Internal Medicine Koshi Hospital Biratnagar Nepal; ^4^ Department of Ophthalmology, Maharajgunj Medical Campus Tribhuvan University Institute of Medicine Maharajgunj Kathmandu Nepal; ^5^ Department of Internal Medicine National University Hospital Singapore Singapore

**Keywords:** ALS, amyotrophic lateral sclerosis, biomarker, OCT, optical coherence tomography, retinal nerve fiber layer

## Abstract

**Introduction:**

Increasing evidence suggests Amyotrophic Lateral Sclerosis (ALS) as a widespread pathological process comprising nonmotor features like fatigue, mild sensory symptoms, cognitive decline, and visual impairment. Measurements of retinal nerve fiber layer (RNFL) thickness using Optical Coherence Tomography (OCT) may correlate with the neurodegeneration associated with ALS. In addition to RNFL thickness, other OCT parameters have been explored in the context of diagnosing ALS and predicting disease severity. In this study, we explore the possibility that OCT parameters of patients with ALS may differ significantly from those of healthy controls and thus serve as biomarkers for the disease and its progression.

**Materials and methods:**

Between 2010 and 2021, the PubMed and EMBASE databases were examined for English language literature. ALS severity was assessed using the revised ALS functional rating scale (ALSFRS‐R). The pooled mean differences in RNFL thickness between ALS patients and controls were calculated using the Standard Mean Difference (Hedges's *g*) with a 95% confidence interval (CI) in STATA software version 16.

**Results:**

Eleven studies were reviewed for data collection. RNFL thickness was not statistically significantly different between ALS patients (*n* = 412) and controls (*n* = 376) (Hedges's *g* = –0.22; 95% CI: –0.51 to 0.07, *I* ^2^ = 73.04%, *p* = .14). However, the thickness of inner nuclear layer was significantly different between ALS patients and controls (Hedges's *g* = –0.38; 95% CI: –0.61 to 0.14, *I* ^2^ = 14.85%, *p* = .00).

**Conclusion:**

Our meta‐analysis found that RNFL thickness as a whole or by individual quadrants was not significantly different between ALS patients and controls while the inner nuclear layer (INL) was substantially thinner.

## 1 INTRODUCTION

Amyotrophic Lateral Sclerosis (ALS) is a neurodegenerative disorder that causes progressive upper and/or lower motor neuron dysfunction. ALS is a devastating and incurable disease. Death often occurs within 3 years of symptom onset in up to 70% of patients (Hübers et al., 2016). It is a diagnosis of exclusion, and diagnosis is often delayed as there exists no specific test that can confirm the disease (Marin, 2019). Recently, several biomarkers have been studied, but none has been implemented in clinical practice yet (Bakkar et al., 2015). Thus, there is a pressing need to find effective investigations that can aid in accurate diagnosis, stratification and monitoring the progress in patients with ALS.

Traditionally, ALS has been considered a pure motor system disease. However, there is an increasing volume of evidence supporting the involvement of other nonmotor systems including the eyes. Given the common embryological origin of retinal cells and neurons of the central nervous system, neurodegenerative conditions that affect the brain and spinal cord may affect retinal cells as well (London et al., 2013; Mancino et al., 2019). Optic coherence tomography (OCT) is a noninvasive imaging test used to obtain high‐resolution images of the retina originally designed to diagnose ophthalmologic diseases. Some studies used OCT as a way of detecting early glaucoma before the development of visual field defects. Now, the uses for OCT are expanding, and OCT parameters are being considered in ALS patients as a diagnostic and prognostic tool (Mukherjee et al., 2017).

Measurements of retinal nerve fiber layer (RNFL) thickness using OCT may correlate with the neurodegeneration associated with ALS. In addition to RNFL thickness, other OCT parameters have been explored in the context of diagnosing ALS and predicting disease severity. Such parameters include retinal layers other than RNFL, whole retinal thickness and macular thickness. In this study, we explored the possibility that OCT parameters of patients with ALS differ significantly from those of healthy controls and thus serve as a potential diagnostic marker for the disease and its progression.

## 2 METHODOLOGY

This systematic review and meta‐analysis are being reported in accordance with PRISMA (Preferred Reporting Items for Systematic Reviews and Meta‐Analyses), using the PRISMA checklist and flow diagram for developing the paper format (Liberati et al., 2009). The first stage in conducting this review was to formulate the research topic. The purpose of this study was to determine whether OCT parameters in ALS patients differ significantly from those in healthy controls. Following that, we developed the study selection criteria, including inclusion and exclusion criteria.

### 2.1 Study inclusion and exclusion criteria

The inclusion criteria were as follows:

- Study type(s): Eligible studies to be included in review were prospective or retrospective studies published in any language.
- Study cases(s): Subjects with ALS of any age, gender, or nationality whose retina was evaluated using OCT were eligible.
- Study control(s): Subjects of any age, gender, or nationality without ALS and other diseases are known to cause retinal thinning, whose retina was evaluated using OCT were eligible.
- Objective outcome(s): Studies should at least compare RNFL thickness between cases and controls. Additional outcomes included but not mandatory were (1) quadrant specific RNFL thickness, (2) thickness of retinal layers other than RNFL, (3) macular thickness, and (4) whole retinal thick ness.
- Study result (s): Studies providing enough data for calculations of mean difference of RNFL thickness between cases and control and its 95% confidence interval were included.

The exclusion criteria were as follows:

- Case reports and case series with ≤5 cases
- Animal studies
- Autopsy studies of the retina in ALS patients
- Review articles
- Studies not reporting our primary outcome

### 2.2 Methods of search

Between 2010 and 2021, the PubMed and EMBASE databases were examined for English language literature. A database search was conducted using Boolean logic, and the Boolean search operators “AND” and “OR” were utilized to connect search words. The following search strategy was used in PubMed: ((“Amyotrophic Lateral Sclerosis”[MeSH Terms] OR “ALS”[All Fields] OR “Motor Neuron Disease”[All Fields] OR “Lou Gehrig Disease”[All Fields]) AND ((“tomography, optical coherence”[MeSH Terms] OR “OCT”[All Fields]) AND (“Retinal Nerve Fiber Layer”[All Fields] OR The following search technique was used in EMBASE: (‘Als’ OR'motor neuron disease'/exp OR ‘amyotrophic lateral sclerosis’/exp OR ‘Lou Gehrig disease’) AND (‘optical coherence tomography’/exp OR ‘oct’) AND (‘retinal nerve fiber layer thickness’/exp OR'retinal nerve fiber layer'/exp OR ‘rnfl’ [humans]/lim Additionally, [2010–2021]/py AND [english]/lim. The search approach in full is included in the supplementary file, Appendix 1. Google Scholar and the China National Knowledge Infrastructure (CNKI) databases were used to conduct a search for foreign language and gray literature. Additionally, the search was expanded to encompass conference proceedings published in journals, preprint servers, and thesis repositories. We combed through the reference lists of each included paper in order to uncover additional potentially relevant material.

### 2.3 Selection of studies

All studies that were shortlisted were then imported into the Mendeley collection, and duplicates were deleted as necessary. A subsequent manual check was performed to remove any remaining duplicates. Papers were initially reviewed separately by two reviewers (GN and SK) for title, keywords, and abstract, and then confirmed by a third reviewer (MAC). Articles that passed the initial screening were then thoroughly evaluated by two reviewers (GN and SK). We resolved disagreements between the two major reviewers (GN and SK) on the final study selection by consulting with a third reviewer (MAC). An assessment of population overlap was undertaken on the basis of authorship, hospital environment, and recruitment time. In cases of overlap, we included papers with a higher quality or bigger sample size.

### 2.4 Extraction of data

Two independent investigators (GN and SK) extracted data using a standardized data extraction form in an Excel spreadsheet (Microsoft Corporation), and the results were collated to complete the following fields: The author, year of publication, study location, study design, number of patients (ALS and controls), age of patients, average RNFL thickness, average RNFL thickness in four quadrants (superior, inferior, temporal, nasal), thickness of other retinal layers (outer nuclear layer, outer plexiform layer, inner nuclear layer, and inner plexiform layer‐ganglion cell layer), whole retinal thickness, and macular thickness were all extracted from ALS patients and healthy controls. When consensus could not be established, a third reviewer (MAK) was consulted to reconcile inconsistencies. If necessary data were omitted, were not given in the article, or were reported in an odd format, the corresponding authors of the individual studies were contacted via email for explanation. In some instances, supplementary material related with the main paper was also examined.

### 2.5 Appraisal of quality

Two investigators (GN and SK) used a consensus process to assess the quality of included studies. The Newcastle‐Ottawa Scale (http://www.ohri.ca/programs/clinical epidemiology/oxford.asp) was used to assess the quality of each study, which were classified into three categories: selection (5), comparability (2), and exposure (3). Two writers evaluated the study separately, and any discrepancies were resolved by conversation with the third author. Studies with a score of 5 or above were considered eligible for inclusion, while those with a score of greater than 7 were regarded to be of high quality. Disagreements were resolved through dialogue with additional reviewers.

### 2.6 Statistical analysis

STATA software version 16 was used for all statistical analysis. The pooled mean differences in RNFL thickness between ALS patients and controls were calculated using the Standard Mean Difference (Hedges's *g*) with a 95% confidence interval (CI). The data were pooled using a random‐effects or fixed‐effect model, and statistical heterogeneity was determined using the *I* ^2^ statistic. When *I* ^2^ reached 50%, meta‐analysis was performed using a fixed‐effect model. When *I* ^2^ was greater than 50%, meta‐analysis was performed using DerSimonian and Laird's random‐effects model. To illustrate the overall weighted mean estimations with 95% CIs, forest plots with 95% CIs were generated. Statistical significance was defined as a *p* < .05.

## 3 RESULTS

### 3.1 Search results and study characteristics

**FIGURE 1:**
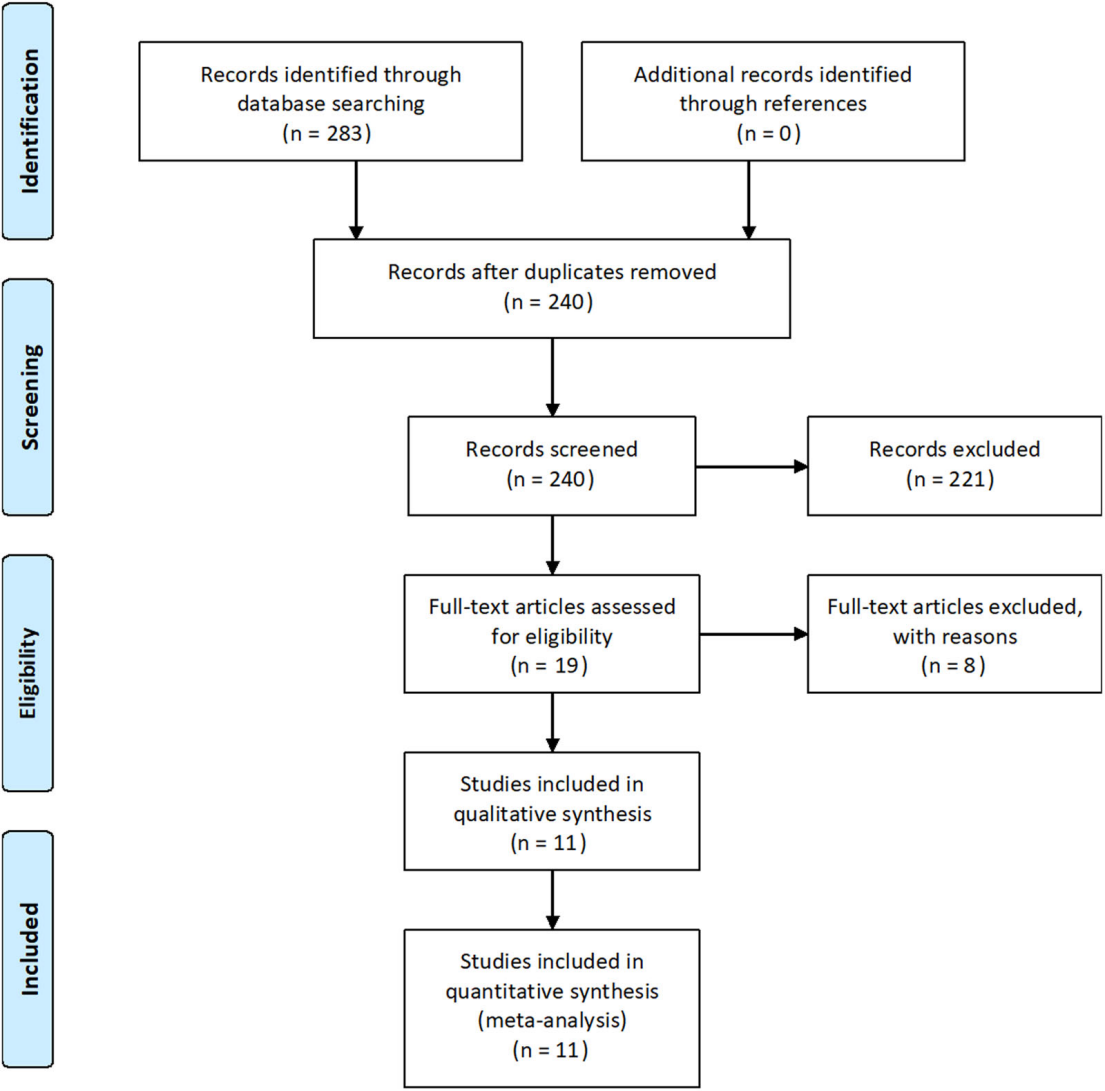
PRISMA flow diagram depicting the flow of information through the different phases of a systematic review

In total, 283 articles were identified after a thorough database search. After the exclusion of duplicates and those not meeting inclusion criteria, 11 studies were reviewed for data collection. Figure 1 shows the results of our literature search and selection. Among 11 studies included, 9 had prospective design whereas two had retrospective design (Abdelhak et al., 2018; Simonett et al., 2016). Four studies were conducted in Germany (Abdelhak et al., 2018; Hübers et al., 2016; Marin, 2019; Ringelstein et al., 2014), two in the United States (Mukherjee et al., 2017; Simonett et al., 2016) and one each in China (Liu et al., 2018), Iran (Rohani et al., 2018), India (Neeraja et al., 2018), and Spain (Rojas et al., 2019). There was one study which was a multicenter study conducted both in the United States and Germany (Roth et al., 2013). The study period among included studies ranged from 2010 to 2017. The total number of ALS patients included in each study ranged from 20 to 70, while the total number of healthy controls ranged from 20 to 126. The mean age of ALS patients ranged from 51 years to 66 years, with mean age > 50 years in all included studies. The total ALS disease duration ranged from 9 months to 43.2 months. ALS severity was assessed using the revised ALS functional rating scale (ALSFRS‐R). The maximum score using this scale is 48, and the score is determined by functional assessment of speech, salivation, swallowing, handwriting, G‐tube dependence, cutting food and handling utensils, dressing and hygiene, turning in bed and adjusting bedclothes, walking, climbing stairs, dyspnea, orthopnea, and respiratory insufficiency. Among the 11 studies included, participants varied widely in disease severity, with mean scores ranging from 28 to 41 at baseline. All included studies measured the RNFL thickness. Many subdivided the retinal nerve fiber layer into quadrants, reporting those measurements, and most studies included findings of the other individual retinal layers. In addition to these parameters, some studies included additional measurements, such as whole retinal thickness and macular thickness. The details of each study are provided in Table 1. The quality assessment of the included studies is provided in the supplementary file, Appendix 2.

**TABLE 1:** Key methodological characteristics of studies included in this systematic review and meta‐analysis

					Participants						
SN	Author	Study period	Study design	Study site	ALS	Control	Mean/Median ALS disease duration (Months)	ALSFRS‐R mean (median)	Sex (F/M) of ALS patient	Mean (median) age of ALS patients	OCT parameters measured
1	Abdelhak 2018	2013–2016	Retrospective	Germany	34	20	12 (7–17)	NA	NA	59 (50–64)	Vessel diameter (IWT, OWT, lumen diameter, mean wall thickness, wall‐to‐lumen ratio) Retinal layer diameter (total macular volume, pRNFL, IPL, INL,GCL, OPL, ONL)
2	Hubers 2015	2012–2014	Prospective	Germany	70	20	12 (2–98)	40 (16−48)	36/35	61 (28−84)	Retinal layer thickness (RNFL, GCL+IPL, INL, OPL, OPL+PR, whole retina)
3	Liu 2017	2016–2017	Prospective	China	51	126	18.46 ± 6.16	39.58 ± 10.41	26/25	55.04 ± 12.52	RNFL thickness by quadrant, macular thickness by quadrant, GCL+IPL
4	Marin 2019	2015–2016	PhD dissertation (prospective)	Germany	34	21	9 (1–72)	Baseline: 41 (26–46) Follow‐up: 37 (16–44)	16/18	66.5 (47–82)	Retinal layer thickness (RNFL, GCL+IPL, INL, OPL, ONL, whole retina)
5	Mukherjee 2017	2013–2014	Prospective	United States	21	21	NA	30 (2–40)	7/14	59 (36–79)	RNFL thickness by sector (global, nasal, superotemporal, inferotemporal, temporal, superonasal, inferonasal) in R and L eyes
6	Neeraja 2018		Prospective	India	25	25	14.07 ± 7.8	31.96 ± 5.99		51.43 ± 11.2 years	RNFL thickness, macular thickness
7	Ringelstein 2014	2010–2012	Prospective	Germany	24	24	22.3 ± 22.57	NA	5/19	61.5 ± 13.0	Thickness of RNFL, GCIP, INL, OPL, ONL, macula
8	Rohani 2018	2015	Prospective	Iran	20	25	14.5	33.1 (3.8)	8/12	56.6 ± 10.7	RNFL thickness by quadrant (superior, inferior, temporal, nasal) in R and L eyes
9	Rojas 2019	2015	Prospective	Spain	Baseline: 20 Follow‐up: 10	38	10.80 ± 5.5	Baseline: 29.50 ± 14.89 Follow‐up: 35.6 ± 14.08	Baseline: 8/12 Follow‐up: 6/4	Baseline: 51.10 ± 9.89 Follow‐up: 51.30 ± 10.02	Ganglion cell complex thickness (superior and inferior quadrants, GCL), RNFL thickness (by quadrant and sector)
10	Roth 2013	NA	Prospective	United States, Germany	76	54	42 ± 34	34 ± 7	26/50	56.1 ± 11.3	Thickness of retinal layer by quadrant, GCIP, INL/OPL, ONL/PRL, RNFL by quadrant
11	Simonett 2016	NA	Retrospective	United States	21	21	43.2 ± 43.4	28.1 ± 12.5	6/15	55.2 ± 10.5	Thickness of RNFL, GCP/IPL, INL, OPL/ONL, IS/OS, RPE

### 3.2 Retinal nerve fiber layer thickness (RNFL)

**FIGURE 2:**
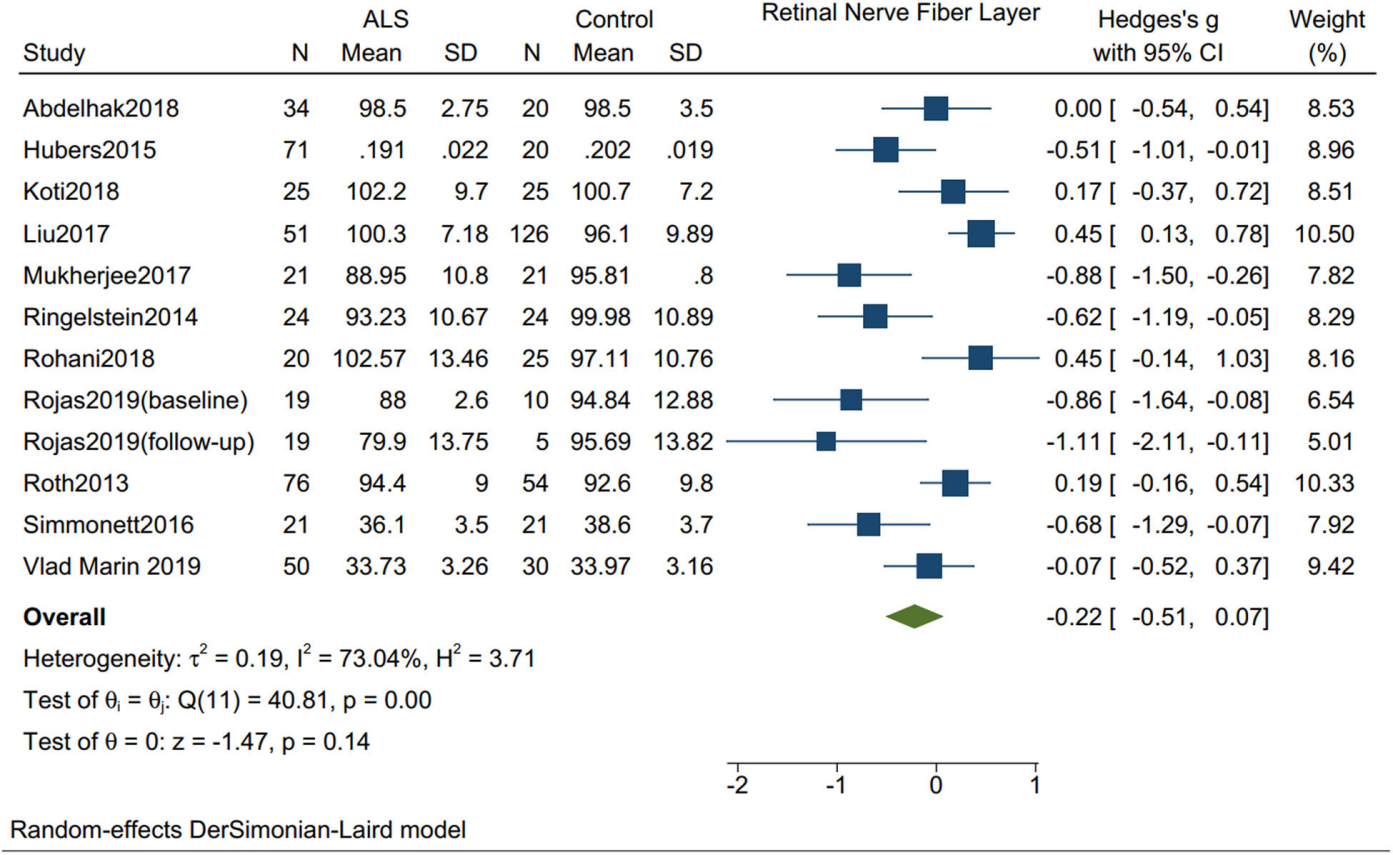
Forest plot with 95% CI showing difference in the thickness of retinal nerve fiber layer between patients with amyotrophic lateral sclerosis and healthy controls. The area of each square is proportional to the study's weight in the meta‐analysis, while the diamond shows the pooled result. The horizontal lines through the square illustrate the length of the confidence interval. The width of the diamond serves the same purpose. The overall meta‐analyzed measure of effect is an imaginary vertical line passing through the diamond

**FIGURE 3:**
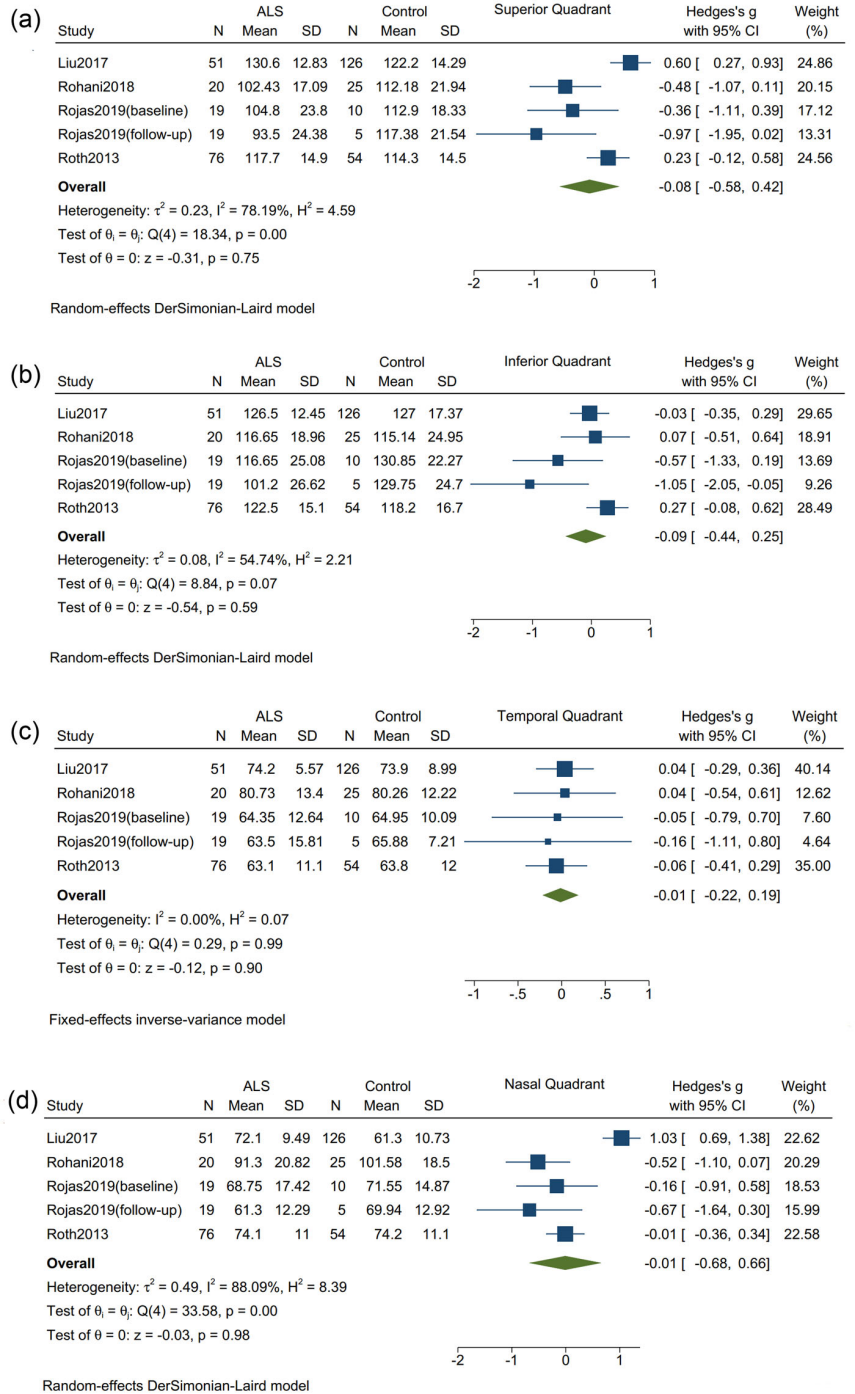
Forest plot with 95% CI showing difference in the thickness of retinal nerve fiber layer between patients with amyotrophic lateral sclerosis and healthy controls. (a) Superior quadrant; (b) inferior quadrant; (c) temporal quadrant; (d) nasal quadrant

RNFL thickness was not statistically significantly different between ALS patients (*n* = 412) and controls (*n* = 376) (Hedges's *g* = −0.22; 95% CI: −0.51 to 0.07, *I* ^2^ = 73.04%, *p* = .14) (Figure 2). The thickness was not significantly different between ALS patients (*n* = 166) and controls (*n* = 215) in the superior quadrant (Hedges's *g* = −0.08; 95% CI: −0.58 to 0.42, *I* ^2^ = 78.19%, *p* = .75, Figure 3a), inferior quadrant (Hedges's *g* = −0.09; 95% CI: −0.44 to 0.25, *I* ^2^ = 54.74%, *p* = .59, Figure 3b), temporal quadrant (Hedges's *g* = −0.01; 95% CI: −0.22 to 0.19, *I* ^2^ = 0%, *p* = .90, Figure 3c), and nasal quadrant (Hedges's *g* = −0.01; 95% CI: −0.68 to 0.66, *I* ^2^ = 88.09%, *p* = .98, Figure 3d).

### 3.3 Thickness of other retinal layers

**FIGURE 4:**
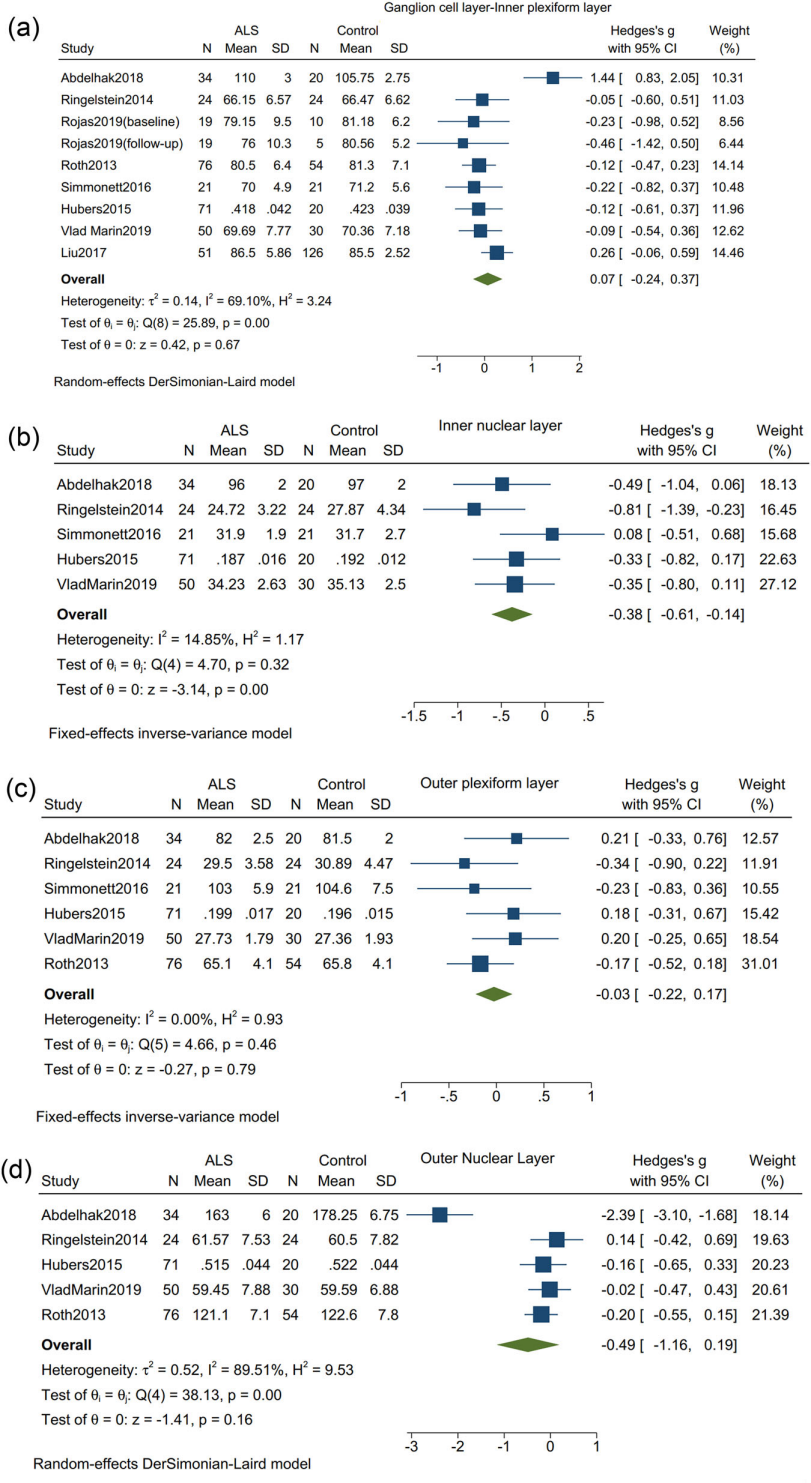
Forest plot with 95% CI showing difference in the thickness of various sub layers of retinal nerve fiber between patients with amyotrophic lateral sclerosis and healthy controls. (a) Ganglion cell layer‐inner plexiform layer; (b) inner nuclear layer; (c) outer plexiform layer; (d) outer nuclear layer

Between ALS patients and controls, no significant difference was seen in the thickness of ganglion cell layer‐inner plexiform layer (Hedges's *g* = 0.07; 95% CI: −0.24 to 0.37, *I* ^2^ = 69.10%, *p* = .67, Figure 4a), the thickness of outer plexiform layer (Hedges's *g* = −0.03; 95% CI: −0.22 to 0.17, *I* ^2^ = 0%, *p* = .79, Figure 4c) and the thickness of outer nuclear (Hedges's *g* = −0.49; 95% CI: −1.16 to 0.19, *I* ^2^ = 89.51%, *p* = .16, Figure 4d). However, the thickness of inner nuclear layer was significantly different between ALS patients and controls (Hedges's *g* = −0.38; 95% CI: −0.61 to 0.14, *I* ^2^ = 14.85%, *p* = .00, Figure 4b).

### 3.4 Macular thickness and whole retinal thickness

**FIGURE 5:**
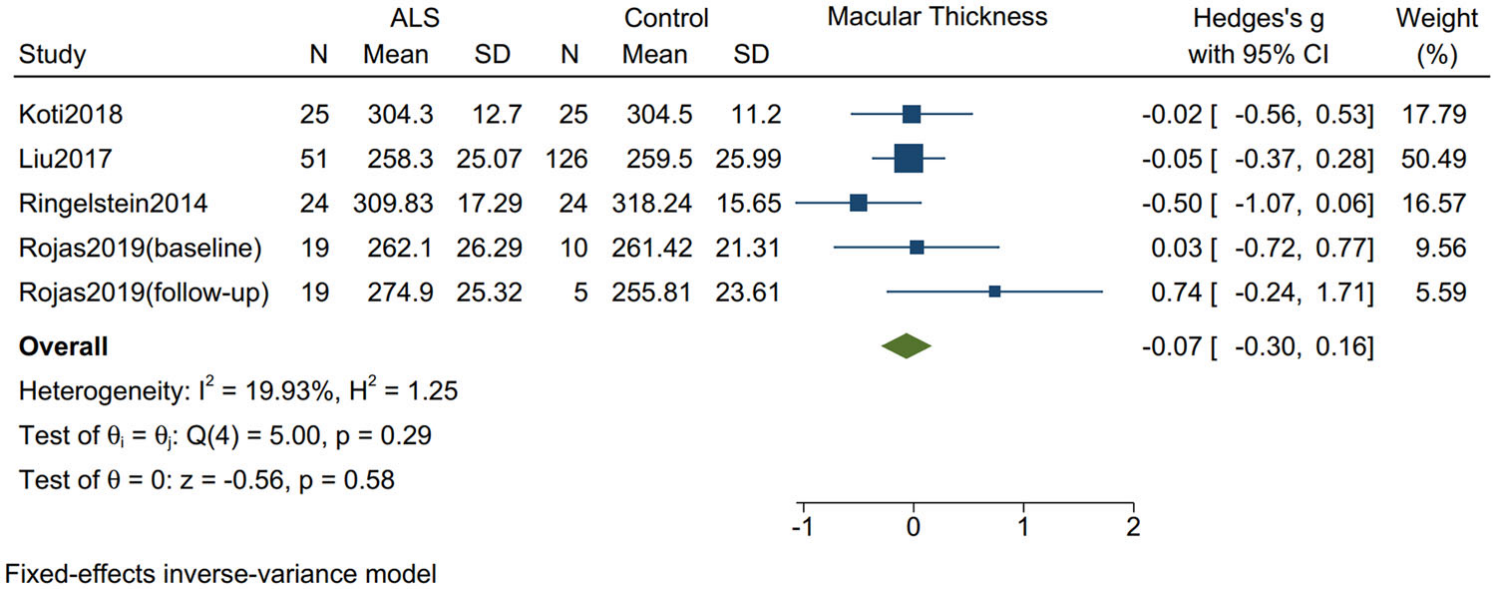
Forest plot with 95% CI showing difference in the macular thickness between patients with amyotrophic lateral sclerosis and healthy controls

**FIGURE 6:**
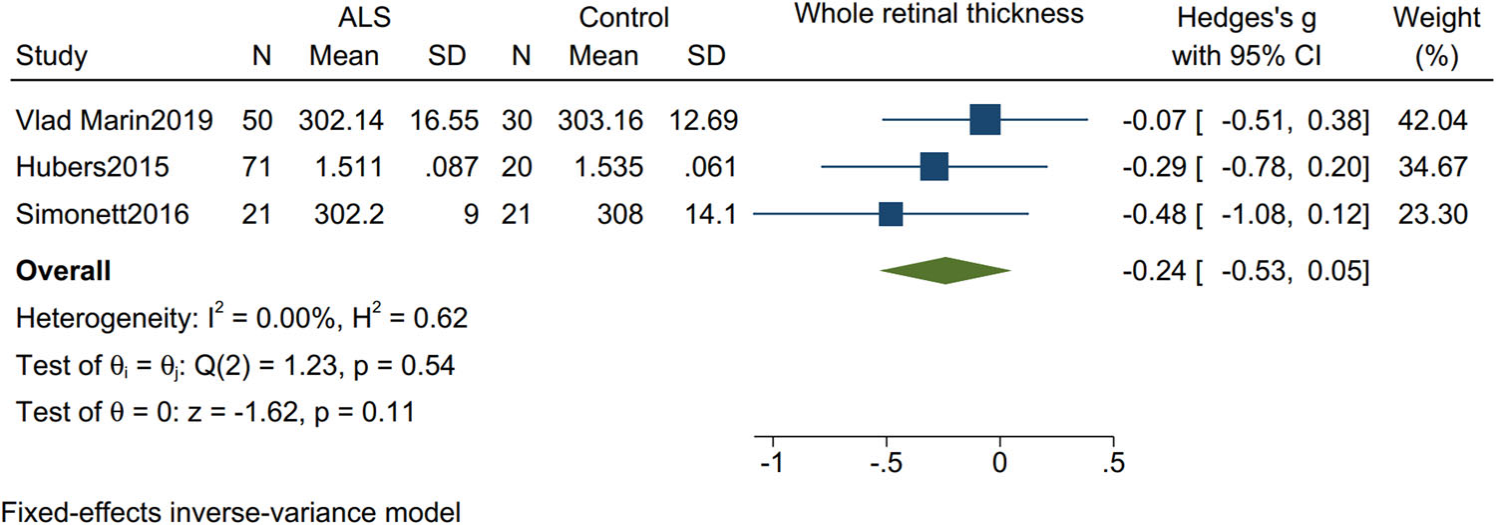
Forest plot with 95% CI showing difference in the whole retinal thickness between patients with amyotrophic lateral sclerosis and healthy controls

There was no significant difference in the thickness of the macula (Hedges's *g* = −0.07; 95% CI: −0.30 to 0.16, *I* ^2^ = 19.93%, *p* = .58, Figure 5) nor the thickness of the whole retina (Hedges's *g* = −0.24; 95% CI: −0.53 to 0.05, *I* ^2^ = 0%, *p* = .11, Figure 6) between ALS patients and controls.

## 4 DISCUSSION

Increasing evidence suggests ALS as a widespread pathological process comprising nonmotor features such as fatigue, mild sensory symptoms, cognitive decline, and visual impairment (Ringelstein et al., 2014). Although visual impairment is not a typical feature of ALS, retinal thinning as evidenced by OCT may reflect an underlying neurodegenerative process and an opportunity to diagnose the condition early before other clinical signs and symptoms arise (Volpe et al., 2015). Anterograde degeneration caused by ganglion cell death in the retina or retrograde degeneration caused by neurodegeneration in the cerebral cortex seem to be two major credible processes that occur in patients with ALS (Mancino et al., 2019; Riancho et al., 2019). It is also worth mentioning that several optic nerve disorders and ALS share common pathogenic mechanisms, which include increased oxidative stress, mitochondrial damage, and axonal transport disorders. Several genes associated with familial forms of ALS such as *OPTN*, *TBK1*, and *ATXN2* have been implicated in chronic primary open‐angle glaucoma, suggesting a common mechanism between both disease conditions (Cirulli et al., 2015). Optineurin is involved in autophagy, vesicular trafficking, and neuroinflammation—key mechanisms that appear to be involved in neurodegenerative conditions including ALS (Toth & Atkin, 2018). Frontotemporal dementia, a disease of the motor neuron disease spectrum, has been associated with thinning of RNFL and ganglion cell layer. Measuring RNFL is widely used to assess optic nerve damage, and its damage has been observed in other neurodegenerative conditions including Parkinson's disease (PD), Alzheimer's disease (AD), and inflammatory neurodegenerative conditions such as multiple sclerosis (Chan et al., 2019; Petzold et al., 2017; Yu et al., 2014). Hence, measuring RNFL and other retinal parameters as biomarkers in ALS makes for a strong case.

In our meta‐analysis, RNFL thickness was not significantly different between ALS patients and controls regardless of the quadrants compared, although individual studies (Hübers et al., 2016; Mukherjee et al., 2017; Rohani et al., 2018; Ringelstein et al., 2014; Simonett et al., 2016) showed significant thinning of the RNFL in ALS patients compared to controls. Similarly, nonsignificant results were obtained for the thickness of the inner plexiform layer, outer plexiform layer, outer nuclear layer, macula, as well as for the total retinal thickness between ALS patients and controls. In ALS patients, however, the thickness of the inner nuclear layer (INL) was very much reduced.

ALS has been linked to superoxide dismutase 1(SOD1), a gene that encodes an enzyme whose function is to remove excess superoxide anion from the cell by converting it to oxygen. Mutant SOD1 is thought to have an unstable structure, resulting in the neurotoxicity seen in ALS and in retinal pathology because of its proteotoxic effect and loss of antioxidant function (Soldatov et al., 2021). In previous murine models, electron microscopy revealed swollen cells and degenerated mitochondria in the INL and outer layer of nuclear cells in transgenic mice (Hashizume et al., 2008). Additionally, in mammalian models of ALS with C9orf72 mutation, specific p62 inclusions were observed in the INL. These disease‐associated cytoplasmic inclusions generated by stress‐induced protein misfolding suggest that the INL is particularly susceptible to the underlying neurotoxicity seen in ALS (Rojas et al., 2020). Interestingly, a recent gene expression study revealed no significant structural retinal changes in FUS murine model of ALS, but found bright inflammatory activation according to the gene expression study (Soldatov et al., 2021).

Aside from ALS, variations in INL thickness have been described in disorders such as multiple sclerosis (MS). In MS, thick INL has been linked to more pronounced inflammation, recent inflammation, or relapsing‐remitting MS (Balk et al., 2019; Cellerino et al., 2019; Kaufhold et al., 2013; Kaushik et al., 2013; Saidha et al., 2012). However, patients with long‐standing primary progressive MS with a low likelihood of relapse, on the other hand, were shown to have considerable INL thinning (Albrecht et al., 2012; Green et al., 2010; Saidha et al., 2011; Schurz et al., 2021). INL thickening seems to be associated with the pathology of inflammation, whilst INL thinning appears to be a retinal pathology associated with MS but independent of optic neuritis. The reasons behind this are unclear, and have yet to be fully elucidated.

OCT‐measured INL thinning appears to be one of the promising indicators of neurodegeneration in ALS. ALS often progress within the initially affected area in the neurological system and progresses to adjacent and contiguous regions. Patients' function and independence deteriorate as the disease progress and the majority of patients die as a result of respiratory failure. The rate at which the disease progresses varies from patient to patient, and the symptoms are determined by the muscles involved (Kiernan et al., 2011). Currently, neuronal damage is mostly quantified clinically by the Revised ALS Functional Rating Scale (ALSFRS‐R), which is severely constrained in that it only partially reflects neurodegenerative damage (Proudfoot et al., 2016). In this setting, OCT offers significant advantages because it is noninvasive, inexpensive, simple, accessible, and has quick modality, which produces standardized quantitative values. This allows for reliable disease activity and treatment response monitoring. Furthermore, OCT may provide a means of measuring subclinical ALS neurodegeneration, aiding in early diagnosis (Gupta et al., 2016).

Our study has several limitations. Our systematic review and meta‐analysis included papers on ALS with varying degrees of severity, and duration of follow‐up following diagnosis. Additionally, except in the study by Mukherjee et al. (2017), the underlying ocular pathology of controls was not screened before inclusion in the study. Without baseline data of the control retina, it is difficult to conclude if ALS is the sole cause of retinal thinning. In future studies, an ophthalmological examination before OCT image acquisition may help any eliminate any confounding retinal pathology. Furthermore, we were not able to perform subgroup analysis based on various forms of ALS, as the individual studies did not report such data. A number of past studies have shown significant differences in retinal thickness between patients with spinal onset ALS and controls but not between patients with bulbar onset ALS and controls (Hübers et al., 2016). Further analysis within subtypes of ALS can bring light to this matter.

## 5 CONCLUSION

Our meta‐analysis found that RNFL thickness as a whole or by individual quadrants was not significantly different between ALS patients and controls. Similarly, the thickness of the inner plexiform, outer plexiform, and outer nuclear layers, as well as macular thickness and total retinal thickness were not significantly different between ALS patients and controls. However, in ALS patients, INL was substantially thinner. Additional research, especially with a larger sample size, a consistent screening process, and follow‐up procedures, are necessary to thoroughly examine the efficacy of measuring retinal thickness in the diagnosis and monitoring of ALS.

## AUTHOR CONTRIBUTIONS

Study concept and design: GN, MAC, SK, RO. Data collection: MAC, PP, SK. Analysis and interpretation of data: GN, SK, JKY. Drafting of the manuscript: GN, YKS, MAC, KP, JKY, and RO. All authors read and approved the final manuscript.

## CONFLICT OF INTEREST

The authors declare no conflict of interest

## FUNDING STATEMENT

None.

### PEER REVIEW

The peer review history for this article is available at: https://publons.com/publon/10.1002/brb3.2741.

## Supporting information

APPENDIX 1 Search strategy used in the current systematic review and meta‐analysis
APPENDIX 2 Quality assessment of the included observational articles

## Data Availability

The data sets of the current study are available from the corresponding author on reasonable request.
