# Supplementary material for: Amyotrophic lateral sclerosis and retinal changes in optical coherence tomography: A systematic review and meta‐analysis
Source: Brain Behav. 2022 Aug 22;12(9):e2741. doi: 10.1002/brb3.2741 (PMC9480919; doi:10.1002/brb3.2741)
Supplement: Supplementary file 1 — APPENDIX 1 Search strategy used in the current systematic review and meta‐analysis APPENDIX 2 Quality assessment of the included observational articles [file BRB3-12-e2741-s001.pdf]

Supplementary Content

Appendix 1: Search strategy used in the current systematic review and meta-analysis.

Appendix 2: Quality assessment of the included observational articles.

This supplementary material has been provided by the authors to give readers additional information.

Appendix 1: Search strategy used in the current systematic review and meta- analysis.

For PubMed

#1: “Amyotrophic Lateral Sclerosis"[Mesh] OR "ALS" OR "Motor Neuron Disease" OR "Lou Gehrig Disease"

#2: "Tomography, Optical Coherence"[Mesh] OR "OCT"

#3: "Retinal Nerve Fiber Layer" OR "RNFL" OR "Macular thinning" OR "Retinal vessel" OR "Retina" OR “Wall Thickness”

#4: #2 AND #3

#5: #1 AND #4

Filters: human subjects, English, Time frame from 2010 to 2021

Final search strategy after filters:

(("Amyotrophic Lateral Sclerosis"[MeSH Terms] OR "ALS"[All Fields] OR "Motor Neuron Disease"[All Fields] OR "Lou Gehrig Disease"[All Fields]) AND (("tomography, optical coherence"[MeSH Terms] OR "OCT"[All Fields]) AND ("Retinal Nerve Fiber Layer"[All Fields] OR "RNFL"[All Fields] OR "Macular thinning"[All Fields] OR "Retinal vessel"[All Fields] OR "Retina"[All Fields] OR "Wall Thickness"[All Fields]) AND ("humans"[MeSH Terms] AND "english"[Language]))) AND ((humans[Filter]) AND (english[Filter]) AND (2010:2021[pdat]))

Total studies: 18

Embase

Search strategy:

#18. #14 AND #15 AND #16 AND [humans]/lim AND [2010-2021]/py AND [english]/lim

#17. #14 AND #15 AND #16

#16. #7 OR #8 OR #9 OR #10 OR #11 OR #12 OR #13

#15. #5 OR #6

#14. #1 OR #2 OR #3 OR #4

#13. 'retinal ganglion cell layer'/exp

#12. retina

#11. 'retinal vessels'

#10. 'macular thinning'

#9. rnfl

#8. 'retinal nerve fiber layer'/exp

#7. 'retinal nerve fiber layer thickness'/exp

#6. oct

#5. 'optical coherence tomography'/exp

#4. 'lou gehrig disease'

#3. 'amyotrophic lateral sclerosis'/exp

#2. 'motor neuron disease'/exp

#1. als

Final search strategy:

(‘Als’ OR ‘motor neuron disease'/exp OR 'amyotrophic lateral sclerosis'/exp OR 'lou gehrig disease') AND ('optical coherence tomography'/exp OR ‘oct’) AND ('retinal nerve fiber layer thickness'/exp OR 'retinal nerve fiber layer'/exp OR ‘rnfl’ OR ‘macular thinning’ OR ‘retinal vessels’ OR ‘retina’ OR ‘retinal ganglion cell layer’/exp) AND [humans]/lim AND [2010-2021]/py AND [english]/lim

Total studies=65

Appendix 2: Quality assessment of the included observational articles.

Study Name	Selection	Comparability	Outcome	Total Score	Included/ Excluded
Abdelhak2018 et al.	3	2	2	7	Included
Hubers2015 et al.	3	2	2	7	Included
Koti2018 et al.	2	2	2	6	Included
Liu2017 et al.	3	2	2	7	Included
Mukherjee2017et al.	3	2	3	8	Included
Reingelstein2014 et al.	3	2	2	7	Included
Rohani2018 et al.	3	2	2	7	Included
Rojas2019 et al.	4	2	2	8	Included
Roth2013 et al.	3	2	2	7	Included
Simonett2016 et al.	2	2	3	7	Included
Vlad Marin2019 et al.	4	2	2	8	Included

Note: Mean scores greater or equal to 5 are included in analysis.
